# Performance comparison of 2D and 3D MRI radiomics features in meningioma grade prediction: A preliminary study

**DOI:** 10.3389/fonc.2023.1157379

**Published:** 2023-03-23

**Authors:** Chongfeng Duan, Nan Li, Xuejun Liu, Jiufa Cui, Gang Wang, Wenjian Xu

**Affiliations:** ^1^ Department of Radiology, The Affiliated Hospital of Qingdao University, Qingdao, China; ^2^ Department of Information Management, The Affiliated Hospital of Qingdao University, Qingdao, China

**Keywords:** meningioma, radiomics, magnetic resonance imaging, 2D features, 3D features

## Abstract

**Objectives:**

The objective of this study was to compare the predictive performance of 2D and 3D radiomics features in meningioma grade based on enhanced T1 WI images.

**Methods:**

There were 170 high grade meningioma and 170 low grade meningioma were selected randomly. The 2D and 3D features were extracted from 2D and 3D ROI of each meningioma. The Spearman correlation analysis and least absolute shrinkage and selection operator (LASSO) regression were used to select the valuable features. The 2D and 3D predictive models were constructed by naive Bayes (NB), gradient boosting decision tree (GBDT), and support vector machine (SVM). The ROC curve was drawn and AUC was calculated. The 2D and 3D models were compared by Delong test of AUCs and decision curve analysis (DCA) curve.

**Results:**

There were 1143 features extracted from each ROI. Six and seven features were selected. The AUC of 2D and 3D model in NB, GBDT, and SVM was 0.773 and 0.771, 0.722 and 0.717, 0.733 and 0.743. There was no significant difference in two AUCs (p=0.960, 0.913, 0.830) between 2D and 3D model. The 2D features had a better performance than 3D features in NB models and the 3D features had a better performance than 2D features in GBDT models. The 2D features and 3D features had an equal performance in SVM models.

**Conclusions:**

The 2D and 3D features had a comparable performance in predicting meningioma grade. Considering the issue of time and labor, 2D features could be selected for radiomics study in meningioma.

**Key points:**

There was a comparable performance between 2D and 3D features in meningioma grade prediction. The 2D features was a proper selection in meningioma radiomics study because of its time and labor saving.

## Introduction

According to the World Health Organization (WHO) classification of tumors of the central nervous system published in 2021 (1), meningioma can be classified into three subtypes: grade 1, grade 2 and grade 3. Grade 1 is low-grade tumors, while grades 2 and 3 are high-grade tumors. The selection of therapy strategy and prognosis are different for low- and high-grade meningioma because of their different biological characteristics (2–4). High-grade meningioma grows aggressively and invades the surrounding structures. Therefore, there is a poor prognosis for patients with high-grade meningioma. As its main treatment, surgical resection is essential for high-grade meningioma (2). Other therapies, such as hormone therapy, cytotoxic chemotherapy or targeted therapy, are complementary choices for tumors that cannot be resected completely (5). Low-grade meningioma grows slowly and rarely shows invasion. Thus, patients with low-grade meningioma usually have a better outcome. Regular follow-up or radiotherapy can be better choices than surgery (6). Given this clinical context, a clear preoperative diagnosis of meningioma and establishing its grade have crucial roles in guiding treatment decisions.

Radiomics has been used widely in recent years (7–9). Many researchers have applied radiomics in the study of meningioma, especially the prediction of meningioma grade (10–15) (**Table 1**). In previous studies, there were two different methods applied to feature extraction: two-dimensional (2D) radiomics features (10, 11) and three-dimensional (3D) radiomics features (12–15). To the best of our knowledge, no study has directly compared the performance of 2D and 3D radiomics features in predicting meningioma grade. In our opinion, this is a very important issue due to the wide use of radiomics in clinical practice.

**Table 1:** Summary of previous studies of radiomics in predicting meningioma grade.

Research groups	Sample size	Modality	Feature type	Result	Conclusion
Duan C et al (10)	188	T1+C	2D	The AUC of radiomics nomogram was 0.952 (95% CI: 0.904–1). The AUC of radiomics nomogram was higher than that of clinical model and radiomics signature with a significant difference.	The radiomics nomogram showed high predictive value and might contribute to the diagnosis and treatment of meningioma.
Duan CF et al (11)	188	T1+C	2D	KNN had the largest net benefit when the threshold probability was <0.50, whereas SVM had the largest net benefit when the threshold probability was >0.50.	The model of SVM and KNN performed better than other models with a larger net benefit.
Park YW et al (12)	194	T1+C, ADC, FA	3D	The best classification system had an AUC of 0.86 (95% CI, 0.74–0.98) in the validation set. The accuracy, sensitivity, and specificity of the best classifier were 89.7, 75.0, and 93.5% in the validation set, respectively.	Radiomics feature-based machine learning classifiers of T1C images, ADC, and FA maps are useful for differentiating meningioma grades.
Lu Y et al (13)	152	ADC	3D	The machine learning classifiers could achieve equivalent diagnostic performance (accuracy = 62.96%) compared to two experienced neuro-radiologists (accuracy = 61.11% and 62.04%). The decision forest achieved the best diagnostic performance in the testing dataset (kappa = 0.64, accuracy = 79.51%).	Decision forest with the ADC value and ADC map-based texture features is a promising multiclass classifier that could potentially provide more precise diagnosis and aid diagnosis in the near future.
Ke C et al (14)	263	T1WI, T2WI, T1+C	3D	The multiparametric MRI model demonstrated the best performance in both the training and external validation cohorts (AUC 0.91, accuracy 89% in the training cohort; AUC 0.83, accuracy 80% in the validation cohort).	Nonbenign meningiomas might be preoperatively differentiated from benign meningiomas by using texture analysis from multiparametric MR data.
Han Y et al (15)	131	T1WI, T2WI, T1+C	3D	The best performance of the radiomics model was obtained by SVM (AUC, 0.956; 95% CI, 0.83–1.00; sensitivity, 0.87; specificity, 0.92; f1-score, 0.90).	The radiomics models are of great value in predicting the histopathological grades of meningiomas, and have broad prospects in radiology and clinics.

AUC, area under receiver operating characteristic curve; CI, confidence interval; KNN, k-nearest neighbors; SVM, support vector machine; ADC, apparent diffusion coefficient; FA, fractional anisotropy.

The purpose of this study was to compare the performance between 2D and 3D features to predict meningioma grade based on enhanced T1-weighted imaging (T1WI). Our aim was to identify the best method and provide a reference for future meningioma radiomics research.

## Materials and methods

### Patients

The ethics review board of our institution approved this retrospective study and waived the requirement for informed consent. We searched for meningioma patients treated at our hospital who accepted surgical resection from January 2012 to January 2022 and selected those meeting the criteria. The inclusion criteria were: (1) the enhanced T1 WI examination was performed one week before surgery, (2) the patients did not receive any treatment before MRI examination, (3) a clear pathology diagnosis and grade of the meningioma was made after surgery, and (4) the image quality was satisfactory for further analysis. The exclusion criteria were: (1) the pathological grade of the meningioma was unclear and (2) the artifacts on the MRI images were severe and they were not suitable for further analysis.

There were 170 high-grade meningiomas meeting the inclusion criteria, including 144 cases of WHO grade 2 meningiomas and 26 cases of WHO grade 3 meningiomas. These 170 cases formed the high-grade group. The number of low-grade meningiomas was more than 1000 cases and far greater than that of high-grade meningiomas. To avoid statistical bias, we randomly selected 170 cases of low-grade meningioma to form the low-grade group to match the high-grade group. Finally, our study included 340 cases of meningioma.

### MRI examination

All patients received enhanced T1 WI examination before surgery. The magnetic resonance imaging (MRI) machines included GE Signa 1.5T/3.0T MRI and Siemens Prisma/Skyra 3.0T MRI. The scan parameters were: TR=1800 ms, TE=10 ms, FOV=25 cm, and slice thickness=5 mm. Every patient was administered 0.1 ml/kg Gd-DTPA before the MRI examination.

### Image preprocessing and tumor segmentation

The images were downloaded as Digital Imaging and Communications in Medicine (DICOM) files from the picture archiving and communications system (PACS) workstation and uploaded to 3D Slicer software (version 4.11, https://www.slicer.org/). Image preprocessing was performed with 3D Slicer to standardize the data across the patients. The “N4ITK MRI bias correction” was performed to remove unwanted low-frequency intensity nonuniformity (16). We used the same “bin width” of 25 to normalize the image intensities from different MRI machines or acquisition protocols. The image was resampled with a 1 × 1 × 1 mm^3^ voxel size to ensure the conservation of scales and directions (17).

**Figure 1:**
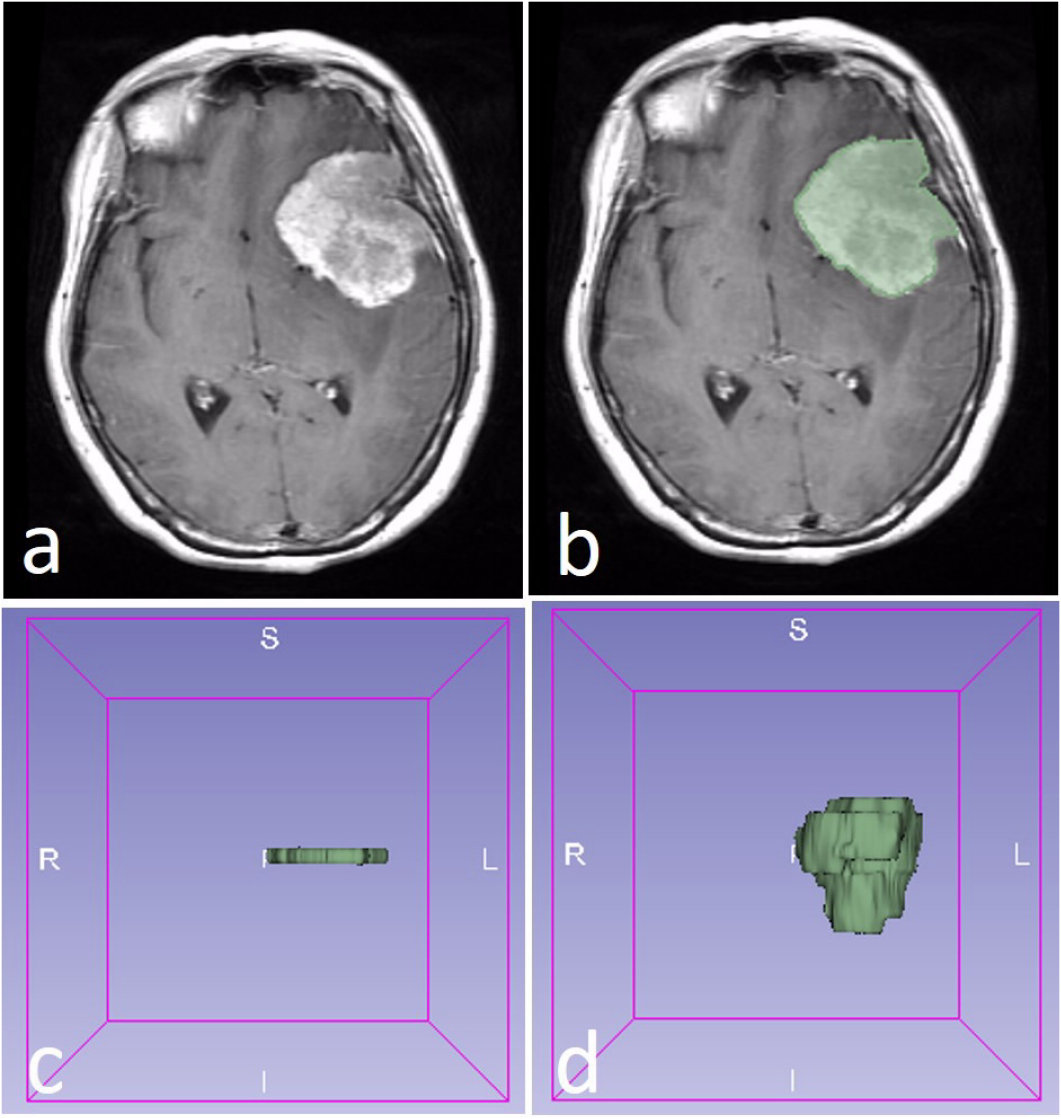
A WHO 2 grade meningioma in a 58-year-old female patient **(A)**. The region of interest (ROI) was drawn along with the edge of the tumor **(B)**. The 2D ROI **(C)** and 3D ROI **(D)** were performed in the tumor.

The ROI of the tumor was drawn with two methods. The 2D ROI was drawn with the largest cross-sectional area of the tumor, while the 3D ROI was drawn with all of the slices of the tumor (**Figure 1**). A 2D ROI and a 3D ROI were made in each tumor.

### Radiomics feature extraction

Radiomics in 3D Slicer was used to extract features for each ROI. There were eight feature groups: first-order, gray-level co-occurrence matrix (GLCM), gray-level dependence matrix (GLDM), gray-level run length matrix (GLRLM), gray-level size zone matrix (GLSZM), neighboring gray-tone difference matrix (NGTDM), shape and wavelet. Finally, we obtained the 2D features and 3D features.

The intraclass correlation coefficient (ICC) was calculated to select the stable features. Forty meningiomas (20 low-grade and 20 high-grade) were selected randomly for ICC calculation. Two neuroradiologists drew the ROI of 40 cases independently. The ICC calculation model was a single rater, absolute agreement, two-way random-effects model (18). We selected the features with high stability (ICC≥0.8) for further analysis.

Spearman’s rank correlation coefficient was analyzed for the selected features to evaluate the relativity with meningioma grade. The features showing relativity (P<0.05) were retained for the next step.

Least absolute shrinkage and selection operator (LASSO) regression was applied to reduce the dimensions of the features after ICC calculation and Spearman correlation analysis. The valuable features were selected for model construction.

### Model construction

**Figure 2:**
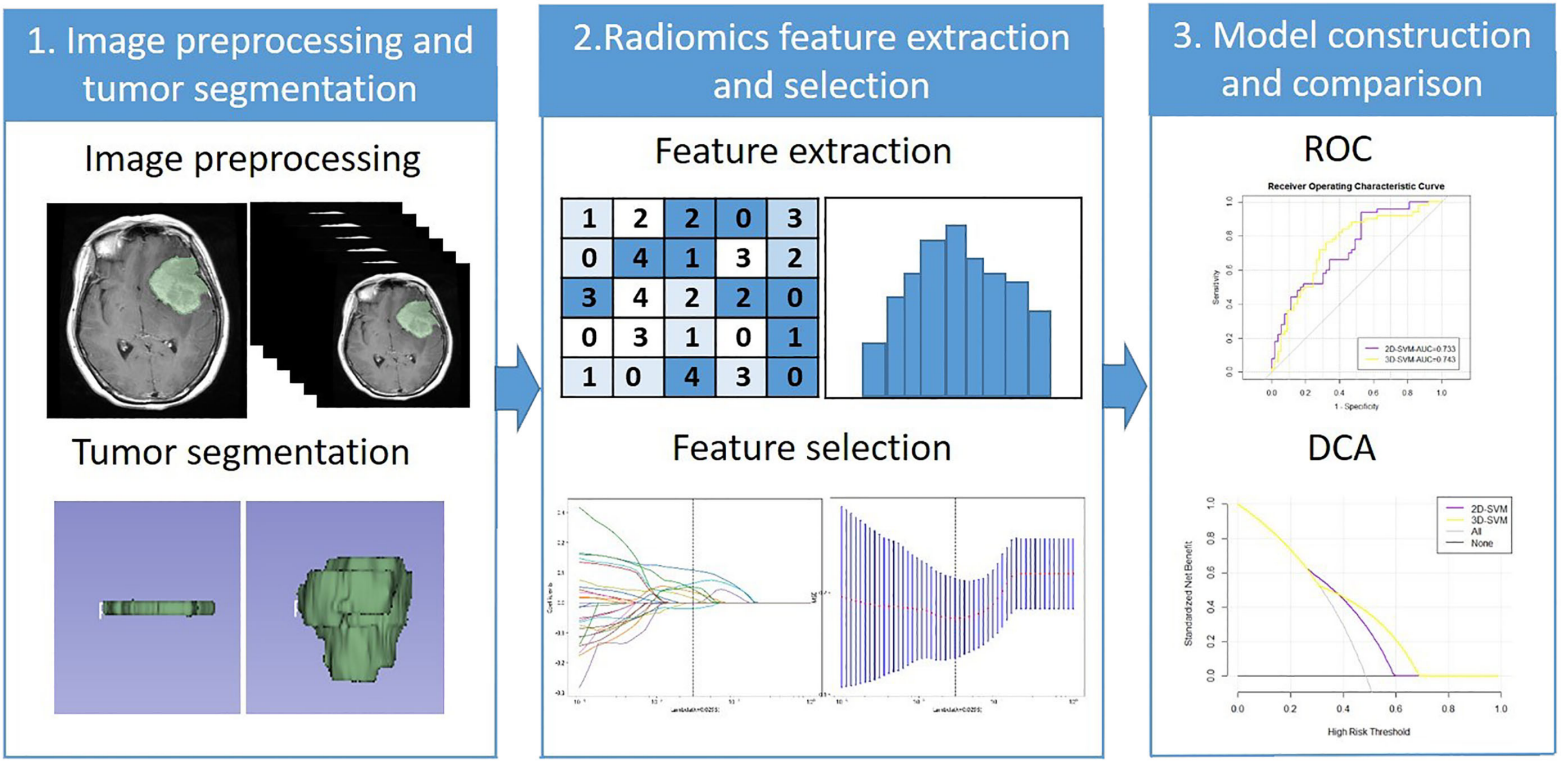
The radiomics workflow. ROC, Receiver operating characteristic; DCA, Decision curve analysis.

The selected features after LASSO were used to construct a predictive model with naive Bayes (NB), gradient boosting decision tree (GBDT), and support vector machine (SVM). The data were divided randomly into a training set (accounting for 70%; 238 cases) and a validation set (accounting for 30%; 102 cases) in each model. Then, 10-fold cross-validation was applied. The model constructed by 2D features and 3D features were defined as the 2D model and 3D model. The ROC curve was drawn, and the AUC of each model was calculated. The Delong text of AUC in each machine learning method was used to evaluate the performance of the two models. The clinical usefulness of the two models was evaluated by calculating the net benefits with decision curve analysis (DCA) (19). The radiomics workflow was shown in **Figure 2**.

### Statistical analysis

R software (https://www.r-project.org/) was used for statistical analysis. The “irr” package, “tidyr and dplyr” package, and “lars” package were applied for ICC calculation, Spearman correlation analysis and LASSO regression. The “e1071” “gbm” package was applied for the NB, GBDT, and SVM model construction. The “pROC” package and “rmda” package were applied for the ROC curve and DCA curve drawing. p<0.05 was considered statistically significant.

## Results

The number of 2D and 3D features was 1143 in total. There were 1068 2D features that showed high stability (ICC≥0.8) and 986 3D features. Among these features, 294 features and 393 features were found with relativity (P<0.05) in the 2D and 3D features. A total of 6 and 7 features were selected by LASSO regression as the 2D and 3D features, respectively. The selected features and their groups are shown in **Table 2**.

**Table 2:** The selected features and their group.

2D Features	Group	3D Features	Group
Coarseness	Wavelet-LLL NGTDM	Maximum3Ddiameter	Original Shape
Coarseness	Wavelet-LLH NGTDM	Sphericity	Original Shape
Coarseness	Wavelet-HHH NGTDM	Imc1	Wavelet-LHL GLCM
Gray level nonuniformity	Wavelet-LHL GLSZM	Run variance	Wavelet-LHH GLRLM
Gray level nonuniformity	Wavelet-LLL GLSZM	Zone entropy	Log-sigma-1-0-mm-3D GLSZM
Skewness	Original First-order	Short run emphasis	Wavelet-HLH GLRLM
		MCC	Wavelet-LLH GLCM

2D, Two dimensional; 3D, Three dimensional; NGTDM, Neighboring gray-tone difference matrix; GLSZM, Gray-level size zone matrix; GLCM, Gray-level co-occurrence matrix; GLRLM, Gray-level run length matrix.

**Figure 3:**
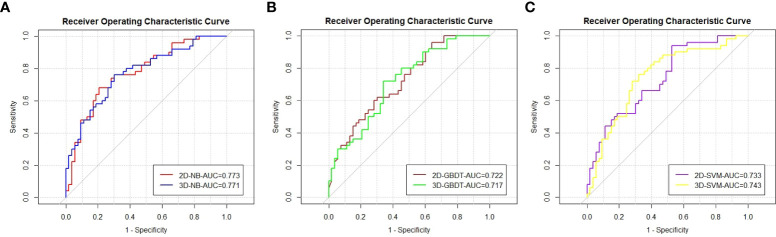
The ROC curves and AUC values of the models by the Naive bayes **(A)**, Gradient boosting decision tree **(B)**, and Support vector machine **(C)**.

The ROC curve and AUC of the 2D and 3D models in each machine learning method are shown in **Table 3** and **Figure 3**. The AUCs of the 2D and 3D models were 0.773 and 0.771, respectively, in NB; were 0.722 and 0.717, respectively, in GBDT; and were 0.733 and 0.743, respectively, in the SVM. There were no significant differences in the AUCs (p=0.960, 0.913, 0.830) between the two models for NB, GBDT, or SVM by Delong text (**Table 3**).

**Table 3:** The AUCs of different methods and results of Delong text.

	2D model	3D model	Z value	P value
NB	0.773	0.771	0.050	0.960
GBDT	0.722	0.717	0.109	0.913
SVM	0.733	0.743	-0.214	0.830

NB, Naive bayes; GBDT, Gradient boosting decision tree; SVM, Support vecto.

**Figure 4:**
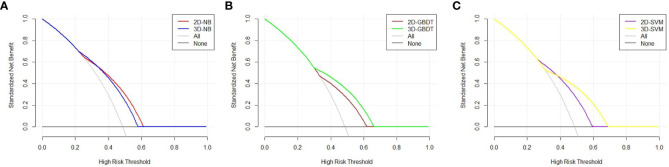
Decision curve analysis of the two models in each machine learning classify. The X axis represents the threshold probability, and the Y axis represents the net benefit. The 2D model has a better performance than 3D model in NB, because 2D model has a larger net benefit than 3D model across most range of the threshold probability **(A)**. The 3D model performs better than 2D model in GBDT due to its larger net benefit in all the threshold probability **(B)**. An intersection of two models at the point of nearly 0.4 can be found in SVM. The 2D model has a better performance at the left of the intersection, while 3D model has a better performance at the right of the intersection **(C)**.

The DCA curve is represented in **Figure 4**. The two models had different performances. The 2D model had a better performance than the 3D model in NB because the 2D model had a larger net benefit than the 3D model across most ranges of the threshold probability. In contrast, the 3D model performed better than the 2D model in GBDT due to its larger net benefit in all threshold probabilities. An intersection of two models at the point of nearly 0.4 can be found in SVM. The 2D model had a better performance to the left of the intersection, while the 3D model had a better performance to the right of the intersection. The models constructed by NB, GBDT, and SVM with 2D and 3D features performed equally well.

## Discussion

In the present study, we compared the performance of 2D and 3D features for meningioma grade prediction. We found that there was no significant difference in AUC, and they performed equally in DCA by different machine learning methods. To the best of our knowledge, this is the first study to compare the performance of 2D and 3D features for meningioma grade prediction.

Previous studies aiming to predict meningioma grade applied different methods for feature extraction. Either 2D or 3D features were used in the previous studies. Duan CF et al. (11) used 2D features to compare different models for predicting meningioma grade. In their study, seven models constructed by 2D features performed well with a high AUC (all>0.80), and SVM and KNN performed better than the other models with an AUC of 0.88 and a larger net benefit in the DCA curve. Duan C et al. (10) first constructed a radiomics nomogram to predict meningioma grade with 2D features. Their radiomics nomogram had a good predictive value. The AUC (0.95) of the radiomics nomogram was significantly higher than that of the other models (p<0.05).

Compared to 2D features, 3D features may be used more widely in meningioma studies. Park YW et al. (12) applied 3D features to machine learning classifiers for meningioma grade and subtype prediction. The most valuable predictive classifier had an AUC of 0.86. Hu J et al. (20) found that a radiomic model based on multiparametric MRI 3D features efficiently predicted meningioma grade. The best model achieved AUCs of 0.84 and 0.81 without or with subsampling, respectively. There were also other studies using 3D features (21, 22). Ugga L et al. (23) argued that adequate standardization of radiomics in meningioma was necessary in future study. Although 2D or 3D features were used in previous studies, their relative performances remained unknown before this study.

Some studies have compared the performance of 2D and 3D features in other diseases (24–27). Shen C et al. (24) found that 2D features (AUC=0.755, validation cohort) had a better performance than 3D features (AUC=0.663, validation cohort) in the prognostic ability of non-small cell lung cancer. Yang G et al. (25) showed that 2D CT texture analysis signature performed better than 3D in predicting lymphovascular invasion in lung adenocarcinoma patients. The AUC of the 2D signature (0.938) was significantly higher than that of the 3D signature (0.753) (P < 0.001). Arefan D et al. (26) reported that the AUC difference between 2D and 3D analysis was not statistically significant when they used MRI radiomics to distinguish axillary lymph node status breast cancer patients. Meng L et al. (27) found that 2D radiomics features had a comparable performance compared with 3D features in characterizing preoperative gastric cancer. In these previous studies, 2D features had a better or equal performance than 3D features.

Researchers have tried to explain the possible mechanisms for the results of the above studies. Shen C et al. (24) gave two reasons for their result. First, it was difficult to conform the consistent resolutions of the CT images and the same transverse plane resolutions in retrospective studies. Second, there may be a slight deviation of the original definition of 3D feature calculation, especially in MRI or PET images because of their thicker layers. Yang G et al. (25) explained that it was mainly related to the technical method of feature calculation. Arefan D et al. (26) considered that a representative slice in 2D features could capture most of the characteristics of the tumor conveyed in the 3D features. Meng L et al. (27) argued that there was more noise in 3D features than 2D features, although 3D features were considered to contain more information. The noise suffered from two aspects: multiple vague lesion boundaries and the higher susceptibility to different thicknesses in 3D features.

In the present study, we found that 2D features had a comparable performance with 3D features. In our opinion, the reasons were similar to those in previous studies. We used the largest cross-sectional area of the tumor to draw the ROI and extracted 2D features. The largest cross-section slice could represent most of the information of the tumor, including size, shape, signal intensity, homogeneous or heterogeneous enhancement, and so on.

The 3D features did not present a better performance than 2D features for several reasons. The routine slice thickness was 5 mm, which is widely used clinically. This slice thickness is relatively high. Therefore, it was difficult to ensure that the ROI was 3D in the true sense. Generally, the so-called 3D ROI might just be a composite of multi2D ROIs. On the other hand, 3D features could suffer from more noise with the increase in slices, especially in tumors with indistinct edges. For example, if a 3D ROI had ten slices, the radiologist would have to draw the tumor edge ten times. However, the radiologist merely drew the edge one time in the 2D ROI. There was more interference from the surrounding structures in the 3D ROI.

This study had some limitations. First, the nature of a retrospective study could lead to selection bias and accuracy overestimation of the performance. Second, the sample capacity of high-grade meningioma was larger than that in most previous studies. The results of the present study need further validation in a larger sample. Third, there was no external validation because of the lack of data from other research centers or hospitals. We will add external validation and a multicenter study in the future.

## Conclusion

The present study compared the predictive value of 2D and 3D radiomics features for meningioma grade. The 2D features had a comparable performance with 3D features. Given the time and labor savings, 2D features can be used in meningioma radiomics studies. Our results provide a reference for future radiomics studies of meningioma.

## Data availability statement

The raw data supporting the conclusions of this article will be made available by the authors, without undue reservation.

## Ethics statement

The studies involving human participants were reviewed and approved by The Affiliated Hospital of Qingdao University. Written informed consent for participation was not required for this study in accordance with the national legislation and the institutional requirements.

## Author contributions

CD: Conceptualization, methodology, software. NL: Data curation, writing- original draft preparation. XL: Software, validation. JC: Supervision. GW: Visualization, investigation. WX: Writing- reviewing and editing. All authors contributed to the article and approved the submitted version.
